# Spore-forming *Clostridia* as overlooked determinants of microbial risk in wastewater reuse systems

**DOI:** 10.1128/aem.01000-26

**Published:** 2026-09-01

**Authors:** Daniel N. Miller, Lisa M. Durso

**Affiliations:** 1 USDA ARS, Agroecosystem Management Research Unit 57654 , Lincoln, Nebraska, USA; 2 (retired) USDA ARS, Agroecosystem Management Research Unit, Lincoln, Nebraska, USA; Michigan State University, East Lansing, Michigan, USA

**Keywords:** wastewater reuse, *Clostridium*, antibiotic resistance

## Abstract

Using treated municipal wastewater for crop irrigation is a key strategy to combat drought-induced water scarcity. However, current wastewater reclamation standards systematically underestimate risks from spore-forming pathogens. As highlighted in a recent minireview by A. Mrozinski, C. Le Maréchal, and E. Topp in *Applied and Environmental Microbiology* (92:e00173-26, 2026, https://doi.org/10.1128/aem.00173-26), *Clostridioides difficil*e and *Clostridium perfringens* survive conventional disinfection, persist indefinitely in agricultural soils, and harbor critical antibiotic resistance genes. To safeguard the food supply and protect public health, regulatory frameworks must shift from relying solely on standard vegetative bacterial indicators and include monitoring resilient, spore-forming pathogens.

## COMMENTARY

## WATER REUSE AND THE RISK OF PATHOGENETIC SPORE FORMERS

Growing evidence indicates that global agriculture has entered a period of severe water scarcity, increasingly described by researchers as a form of “water bankruptcy.” Because agriculture accounts for >70% of global freshwater use, food security/food-production systems become more vulnerable to tightening water constraints. As freshwater scarcity grows, attention is turning toward treated wastewater (TWW) as a practical resource for crop irrigation to help meet water shortages (1), but it raises concerns over the spread of human and foodborne pathogens. While the history of wastewater (and its reuse for agriculture) can be traced back to the Bronze Age with the rise of ancient civilizations (2), pathogen contamination in wastewater has inadvertently spread pathogens through communities, serving as a major vector of human and animal mortality over the centuries. Certainly, advances in wastewater treatment processes have effectively reduced many potential human and environmental health issues associated with wastewater (3), but there is a growing recognition that certain pathogens may survive the wastewater treatment process and pose a risk to future agricultural production (4).

**Fig 1:**
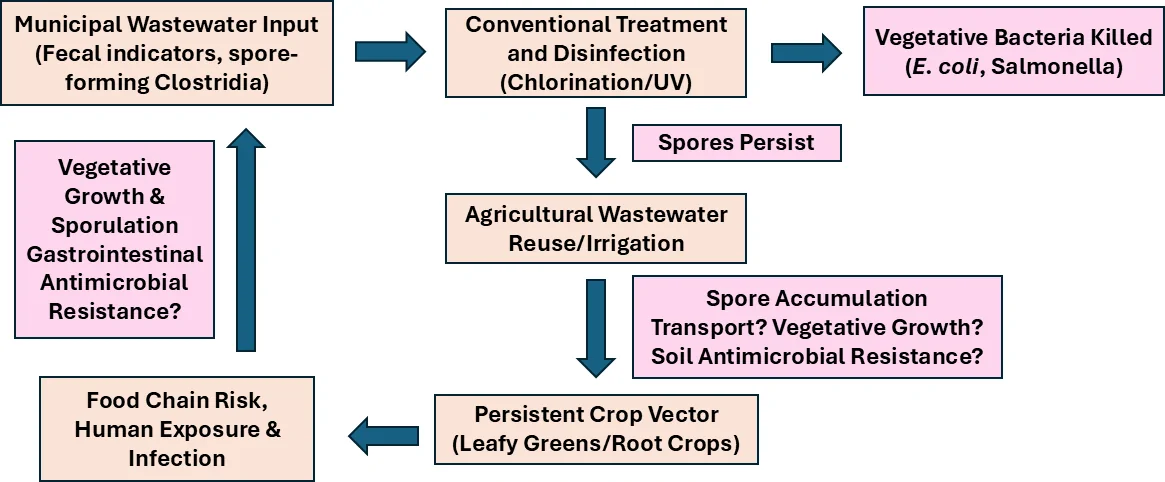
Potential risks for environmental accumulation and human exposure of spore-forming pathogens with current wastewater treatment practices with crop irrigation reuse of the treated wastewater. Typical fecal indicator microorganisms are effectively managed, whereas spore-forming pathogens may accumulate and potentially serve as a conduit for soil and gut antimicrobial resistance elements.

A recent minireview by Mrozinski et al. (5) specifically addresses this concern and concisely summarizes a growing body of published work that argues that persistent, spore-forming pathogens like *Clostridioides difficile* and *Clostridium perfringens* are an important and under-recognized threat that current wastewater treatment practices may not effectively remove. Based upon substantive differences in biology from current indicator microorganisms, these spore-forming pathogens, which are readily isolated from raw (and treated) sewages (6), demonstrate a substantial advantage to survive current wastewater treatment and disinfection processes (7, 8) that effectively reduce other common indicator microorganisms like fecal coliforms, *Escherichia coli*, and Salmonella (Fig. 1). Following TWW reuse for agriculture (irrigation), the spores of *C. difficile* and *C. perfringens* would persist and potentially accumulate in soils with repeated additional TWW applications and potentially transport to nearby environments (9, 10). Certain crops grown close to the ground or within the soil, such as leafy greens or root crops, may be particularly at risk for contamination, with spores that survive initial washing during food processing, contaminate food production systems, and eventually expose and infect humans or other animals (11–13).

Mrozinski et al. (5) argue that an additional important concerning risk associated with *C. difficile* and *C. perfringens* is the diversity of virulence elements and antibiotic resistance genes (ARGs) in soil and gastrointestinal isolates and a potential for horizontal gene transfer (14, 15). While many TWW studies characterize and quantify ARGs in gram-negative bacteria, they seldom consider gram-positive spore-forming anaerobes as long-term reservoirs (16, 17). The longevity of the spore-forming *Clostridia* combined with their dual-habitat lifestyle in both the soil environment and the gut enhances their role as reservoirs of antimicrobial resistance genes and agents of potential horizontal gene transfer. By highlighting the high prevalence of mobile elements in *C. difficile* and *C. perfringens*, Mrozinski et al. (5) contend that these organisms could act as a key nexus for horizontal gene transfer between soil and gut microbiomes (18).

Most reclaimed-water guidelines and quantitative microbial risk assessments do not assess spore-forming *Clostridia*, and existing policies involving TWW reuse for agriculture may be insufficient to assess and mitigate their risks both in Europe and across the globe. Clostridium species have rarely been integrated into food-chain exposure models (19), and although *C. perfringens* testing is currently part of standard TWW microbiology methods, it is often treated as a generic indicator organism rather than an agent or primary agents of concern. There is growing recognition that *Clostridioides difficile* and *Clostridium perfringens* are not merely isolated clinical threats or members of a useful group of TWW indicators, but rather, they represent part of the environmental reservoir of antimicrobial resistance and virulence that remain a challenge for current water-reuse guidelines that largely rely on vegetative indicators (20).

## PROPOSED SOLUTIONS: CALL TO ACTION FOR SCIENTIFIC AND POLICY COMMUNITY

Mrozinski et al. (5) provide a compelling foundation for rethinking how spore-forming pathogens are assessed and addressed in wastewater-reuse systems. *Clostridioides difficile* and *Clostridium perfringens* are persistent, mobile, and adaptable—a risk that current monitoring frameworks do not adequately capture. Moving forward, the scientific community has a clear need to develop surveillance tools specific to toxigenic *Clostridia* pathogens, refine risk-assessment models, and generate the mechanistic data needed to inform evidence-based policy. Regulators and water-resource managers not only in Europe but across the globe need these insights to strengthen reclaimed-water guidelines and incorporate spore-forming pathogens into routine monitoring. By acting on these priorities, researchers and policymakers can help ensure that wastewater reuse advances agricultural efforts without compromising food safety or human and animal health.
